# Urinary TBARS as a Non-Invasive Proxy of Plasma Lipid Peroxidation in Essential Hypertension: A Translational Study on Vascular Oxidative–Inflammatory Burden

**DOI:** 10.3390/antiox15070861

**Published:** 2026-07-09

**Authors:** Antón Cruces-Sande, Néstor Vázquez-Agra, Óscar Seoane-Casqueiro, Emma López-Prado, Estefanía Méndez-Álvarez, Ramón Soto-Otero, Antonio Pose-Reino, Álvaro Hermida-Ameijeiras

**Affiliations:** 1 Laboratory of Neurochemistry, Department of Biochemistry and Molecular Biology, Faculty of Medicine, University of Santiago de Compostela, 15782 Santiago de Compostela, A Coruña, Spain; estefania.mendez@usc.es (E.M.-Á.); ramon.soto@usc.es (R.S.-O.); 2 Health Research Institute of Santiago de Compostela (IDIS), Travesía da Choupana s/n, 15706 Santiago de Compostela, A Coruña, Spain; oscar.seoane.casqueiro@sergas.es (Ó.S.-C.); antonio.pose.reino@sergas.es (A.P.-R.); alvaro.hermida@usc.es (Á.H.-A.); 3 School of Industrial Engineering, University of Vigo (UVigo), 36310 Vigo, Pontevedra, Spain; emma.lopez.prado@sergas.es

**Keywords:** essential hypertension, oxidative stress, lipid peroxidation, TBARS, malondialdehyde, urinary biomarkers, plasma biomarkers, redox biomarkers, vascular inflammation, non-invasive assessment

## Abstract

Background/Objectives: Lipid peroxidation is a relevant oxidative–inflammatory mechanism in essential hypertension and cardiovascular disease. Plasma thiobarbituric acid reactive substances (TBARS), commonly reported as malondialdehyde-equivalent values, provide an operational index of lipid peroxidation-related aldehydic reactivity, but blood-based assessment is limited by venipuncture and preanalytical handling requirements. Urine is an attractive non-invasive matrix for redox biomarker development, although whether urinary TBARS reflect plasma lipid peroxidation in hypertensive patients remains insufficiently characterized. This study aimed to evaluate whether matrix-specific normalization—total cholesterol for plasma TBARS and creatinine for urinary TBARS—reveals a measurable intra-individual relationship between these matrices in essential hypertension. Methods: In this paired observational study, plasma and urine samples were obtained from 39 treated patients with essential hypertension under standardized fasting conditions. TBARS were quantified using a colorimetric thiobarbituric acid reaction assay. Plasma TBARS were normalized to total cholesterol and expressed as TBARSp, while urinary TBARS were normalized to creatinine and expressed as TBARSu. Associations were assessed using Spearman’s rank correlation, exploratory receiver operating characteristic (ROC) analyses based on internally derived plasma TBARS percentile thresholds, and Bayesian bootstrap inference. Results: Cholesterol-normalized plasma TBARS and creatinine-normalized urinary TBARS showed a moderate-to-strong positive monotonic association (Spearman’s ρ = 0.717, *p* < 0.001). Bayesian bootstrap analysis supported this relationship, with a 95% credible interval of 0.57–0.83 and a Bayes factor > 300 for ρ ≥ 0.5. Urinary TBARS showed exploratory within-cohort discriminatory capacity for identifying elevated plasma TBARS using internally derived thresholds, with an AUC of 0.892 for the median-based classification. Conclusions: Creatinine-normalized urinary TBARS showed a moderate-to-strong association with cholesterol-normalized plasma TBARS in treated essential hypertension. These findings provide hypothesis-generating paired-sample evidence that urinary TBARS may serve as a low-burden, non-invasive proxy of plasma lipid peroxidation-related redox alterations. Further validation in larger and clinically diverse cohorts, ideally including more specific lipid peroxidation markers and renal-function-aware analyses, is required to define their translational and clinical utility.

## 1. Introduction

Oxidative stress is a context-dependent biological process in which oxidant production, antioxidant buffering, redox signaling, and irreversible molecular damage interact across spatial and temporal scales [1,2]. In cardiovascular disease, this imbalance is particularly relevant within the vascular compartment, where reactive oxygen species (ROS) contribute to endothelial dysfunction, nitric oxide depletion, vascular remodeling, low-grade inflammation, and lipid peroxidation [3,4,5]. Among the molecular targets of oxidative stress, polyunsaturated fatty acids (PUFAs) are especially vulnerable to radical-mediated chain reactions, generating lipid hydroperoxides and secondary aldehydic products such as malondialdehyde (MDA) and 4-hydroxy-2-nonenal (4-HNE) [6,7,8]. These products may also amplify oxidative–inflammatory signaling through covalent modification of proteins, lipoproteins, and nucleophilic cellular targets [8,9,10].

Although blood-based oxidative stress biomarkers cannot be assumed to reflect every localized tissue process, the vascular compartment represents a biologically relevant setting for plasma redox assessment [11]. In essential hypertension, oxidative stress is generated through mechanisms involving endothelial dysfunction, vascular smooth muscle activation, renin–angiotensin–aldosterone system signaling, mechanical stretch, mitochondrial dysfunction, and NADPH oxidase-derived ROS [3,12]. These processes promote lipid peroxidation in circulating lipoproteins, albumin-bound fatty acids, and vascular wall microenvironments, thereby generating soluble or diffusible aldehydic products that may enter the plasma pool [13,14,15]. Thus, in hypertension, plasma lipid peroxidation biomarkers may provide clinically accessible information on vascular oxidative–inflammatory activity.

Lipid peroxidation can be assessed through several families of biomarkers, including F_2_-isoprostanes, oxidized LDL, 4-HNE adducts, and MDA-related measures [14,16,17,18]. Among these, F_2_-isoprostanes are often considered highly specific indices of lipid peroxidation, whereas MDA and related aldehydes are widely used because they can be measured with relatively simple and scalable analytical approaches [16,17,19]. In cardiovascular research, increased lipid peroxidation markers have been associated with blood pressure, arterial stiffness, carotid intima-media thickness, coronary calcification, endothelial dysfunction, and adverse cardiovascular outcomes [5,14,20,21,22].

The thiobarbituric acid reactive substances (TBARS) assay remains one of the most widely used approaches for estimating MDA-equivalent lipid peroxidation products because it is inexpensive, colorimetric, and compatible with relatively high-throughput workflows [23,24,25]. However, TBARS should be interpreted as an operational measure rather than a molecule-specific quantification of MDA. Under acidic and high-temperature conditions, thiobarbituric acid reacts with MDA and other aldehydic or carbonyl-containing compounds, generating a chromogenic adduct whose absorbance provides a standardized index of TBA-reactive products [23,25,26]. Therefore, TBARS are best understood as a practical proxy of lipid peroxidation-related aldehydic reactivity, particularly useful when analytical simplicity and scalability are priorities.

The limitations of plasma-based oxidative stress assessment have fostered interest in urine as a complementary matrix for redox biomarker development. Urine is non-invasive, easy to collect repeatedly, largely aproteic under physiological conditions, and generally less affected by cellular and enzymatic activity after collection than blood-derived matrices [27,28]. In addition, urinary dilution can be partially corrected through creatinine normalization, facilitating comparison across individuals and sampling conditions [29,30]. From a redox biomarker perspective, urine may provide an integrated excretory signal of systemic oxidative processes while reducing several logistical constraints associated with venipuncture, immediate centrifugation, and cold-chain handling [27,31,32].

However, urinary TBARS should not be considered a simple passive reflection of plasma TBARS. TBA-reactive products detected in urine may derive from circulating lipid peroxidation products after filtration and renal handling, from systemic metabolism or conjugation of reactive aldehydes, and potentially from local renal or urinary tract oxidative processes [27,33,34]. Therefore, establishing whether urinary TBARS track plasma TBARS within the same individuals is a necessary first step before considering them as exploratory non-invasive proxies of vascular lipid peroxidation-related redox alterations. We hypothesized that, in patients with treated essential hypertension, applying biologically grounded matrix-specific normalization—total cholesterol for plasma TBARS and creatinine for urinary TBARS—would reveal a positive intra-individual association between both matrices, supporting urinary TBARS as a low-burden exploratory proxy of plasma lipid peroxidation-related redox alterations.

## 2. Materials and Methods

### 2.1. Design, Setting, and Participants

This was an observational paired-design study conducted at the Hypertension and Cardiovascular Risk Unit of the University Hospital Complex of Santiago de Compostela (CHUS, Santiago de Compostela, Spain) during the first semester of 2024. Patients aged ≥18 years with essential hypertension were consecutively recruited from routine clinical practice. All participants underwent blood and urine sampling during the same on-site clinical visit, under standardized fasting conditions, with simultaneous measurement of plasma and urinary lipid peroxidation-related biomarkers. The primary objective was to evaluate the intra-individual association between cholesterol-normalized plasma TBARS and creatinine-normalized urinary TBARS. Patients were not included if they had secondary hypertension, inability to provide informed consent, incomplete paired plasma–urine sampling, or clinically overt acute intercurrent conditions at the time of sampling, including acute infection, acute inflammatory disease, or recent major cardiovascular events. Stable cardiometabolic comorbidities, including diabetes mellitus, chronic kidney disease (CKD), and established cardiovascular disease, were not exclusion criteria, in order to preserve the real-world nature of the cohort. CKD was defined as an estimated glomerular filtration rate (eGFR) < 60 mL/min/1.73 m^2^. Because each participant served as their own plasma–urine comparison, exclusion criteria were kept minimal; renal function and relevant comorbidities were recorded and specifically considered in the interpretation and sensitivity analyses of the findings.

### 2.2. General Sample Variables

Clinical and demographic variables were collected from all participants, including age, sex, alcohol intake, smoking status, physical activity, body mass index (BMI), blood pressure, renal function, cardiometabolic comorbidities, antihypertensive medication use, and treatment adherence. Alcohol intake was categorized as non-drinker or low-risk drinker, smoking status as no/yes, and physical activity according to the European Society of Hypertension (ESH) recommendations [35]. BMI was calculated as weight divided by height squared (kg/m^2^). Blood pressure was measured according to the STRIDE BP standards endorsed by the ESH [36]. Essential hypertension, diabetes mellitus, chronic kidney disease, and cardiovascular disease were defined according to the corresponding ESH, European Society of Cardiology (ESC), and American Diabetes Association (ADA) consensus documents [35,37,38].

### 2.3. Sample Collection

Blood and urine samples were obtained during the same on-site clinical visit after a 12 h overnight fast, ensuring a minimum interval of 12 h since the last medication intake. Blood was drawn by peripheral venipuncture from the antecubital fossa using the vacuum method, applying less than one minute of tourniquet pressure, into EDTA-containing tubes [24]. Blood samples were immediately kept at 4 °C and centrifuged at 1000× *g* for 10 min at 4 °C. The plasma fraction was collected, aliquoted, and immediately transferred to dry ice prior to storage.

Urine samples were collected on site at the clinical unit, coinciding with blood sampling and under fasting conditions. Rather than relying on urine samples collected at home, on-site fasting spot urine collection was used to reduce preanalytical variability related to uncontrolled storage temperature, transport conditions, delayed processing, and prolonged residence time before centrifugation and freezing. Immediately after collection, urine samples were placed on ice and kept at 4 °C until centrifugation. Urine was collected using vacuum urine biochemistry tubes and centrifuged within 15 min of collection at 1000× *g* for 10 min at 4 °C. The urinary supernatant was collected, aliquoted, and immediately transferred to dry ice prior to storage. Plasma and urine aliquots were stored at −80 °C for no longer than one month before TBARS analysis. This preanalytical workflow follows the same on-site fasting collection and rapid cold-processing strategy previously applied by our group for urinary redox biomarker assessment in essential hypertension [39].

### 2.4. Assessment of Thiobarbituric Acid Reactive Substances in Plasma and Urine

Thiobarbituric acid reactive substances (TBARS) were quantified in plasma and urine using a colorimetric thiobarbituric acid reaction assay adapted from classical protocols [23,24,25]. Under acidic and high-temperature conditions, thiobarbituric acid (TBA) reacts with malondialdehyde (MDA) and other aldehydic or carbonyl-containing compounds to generate a chromogenic TBA-reactive adduct measurable in the visible spectrum. Accordingly, TBARS values were interpreted as an operational index of MDA-equivalent lipid peroxidation-related aldehydic reactivity, rather than as a molecule-specific quantification of MDA.

A 1% TBA solution was prepared using TBA powder (catalog number T5500-25G, ≥98%, Sigma-Aldrich^®^, St. Louis, MO, USA) in 20% acetic acid, adjusted to pH 3.0 with sodium hydroxide. A 10 mM butylated hydroxytoluene (BHT) solution prepared in absolute ethanol was added to minimize in vitro auto-oxidation and artifactual lipid peroxidation during sample processing [25]. Standard calibration was performed using MDA (catalog number 8207560250, ≥99%, Sigma-Aldrich^®^, St. Louis, MO, USA), and calibration curves with coefficients of determination above 0.98 were accepted for analysis.

Plasma, urine, and calibration samples were processed under identical analytical conditions. Reaction mixtures were heated at 90 °C for 30 min, cooled at −20 °C for 10 min, and centrifuged at 4000× *g* for 10 min to remove particulate material and reduce optical interference. Subsequently, 250 µL of each processed sample or calibration standard was transferred to a microplate and measured in quadruplicate using an Asys UVM-340 microplate reader (Biochrom^®^, Cambridge, UK) at 530–540 nm. Results were expressed as MDA-equivalent TBARS concentrations.

Plasma TBARS concentrations were normalized to plasma total cholesterol concentration (TCp) and expressed as nmol/mg TCp [14,40]. Urinary TBARS concentrations were normalized to urinary creatinine concentration (Cru) and expressed as nmol/mg Cru [27,29]. These normalized variables are hereafter referred to as TBARSp and TBARSu, respectively.

### 2.5. Ethics in Research

The study was conducted in accordance with the Declaration of Helsinki and Good Clinical Practice standards. All participants provided written informed consent before inclusion. The study protocol was approved by the Research Ethics Committee of Santiago–Lugo, Spain (protocol code 007/2023; approval date: 25 January 2023).

### 2.6. Statistical Analysis

Statistical analyses were performed using SPSS software, version 22.0 (SPSS Inc., Chicago, IL, USA), following a primarily non-parametric approach because the variables of interest showed non-normal distributions. Sample size was calculated using EPIDAT software, version 4.2 (Dirección Xeral de Saúde Pública, Xunta de Galicia, Santiago de Compostela, Spain), based on the objective of detecting a correlation coefficient of 0.5 between cholesterol-normalized plasma TBARS and creatinine-normalized urinary TBARS, with a 95% confidence level and a statistical power of at least 80% [41].

Variable distributions and extreme observations were inspected before the final inferential analyses. Extreme observations were winsorized to 1.5 times the interquartile range (IQR) above the third quartile and below the first quartile. Winsorized values were then used for the descriptive, correlation, ROC, and Bayesian bootstrap analyses. The absence of missing values in the variables of interest was verified. Descriptive analyses were performed, and results were expressed as number (percentage) for qualitative variables and median (interquartile range) for quantitative variables.

The intra-individual association between TBARSp and TBARSu was evaluated using Spearman’s rank correlation analysis. A logarithmic transformation of both variables was applied only for graphical visualization of the monotonic relationship and was not intended to imply linearity on the original scale. A *p*-value < 0.05 was considered statistically significant. To assess the potential influence of selected clinical and lifestyle factors on the plasma–urine association, sensitivity analyses were performed by recalculating Spearman’s rank correlation after excluding participants with chronic kidney disease, diabetes mellitus, current smoking, or alcohol intake. CKD was defined as eGFR < 60 mL/min/1.73 m^2^. These analyses were reported as Supplementary Materials.

Receiver operating characteristic (ROC) curve analyses were performed to explore the ability of TBARSu to discriminate between lower and higher TBARSp values, using internal plasma TBARS percentile thresholds (P25, P50, and P75) as reference cut-off points. Results were reported as area under the curve (AUC), standard error (SE), *p*-value, 95% confidence interval, sensitivity, and 1−specificity. These ROC analyses were considered exploratory because the thresholds were derived from the internal distribution of the present cohort rather than from externally validated clinical cut-offs and were not intended to define diagnostic thresholds or clinical decision limits.

In addition, a Bayesian bootstrap approach was applied to estimate the posterior distribution of Spearman’s rank correlation coefficient (ρs) between TBARSp and TBARSu [42]. A total of 5000 bootstrap replicates were generated using Dirichlet-distributed weights applied to the original observations. For each replicate, ρs was recalculated using a weighted rank-based covariance formula. From the resulting posterior distribution, a 95% credible interval was derived, and a Bayes factor was calculated to compare the hypotheses H1: ρs ≥ 0.5 versus H0: ρs < 0.5 [42,43,44,45].

## 3. Results

### 3.1. General Characteristics of the Study Population

**Figure 1:**
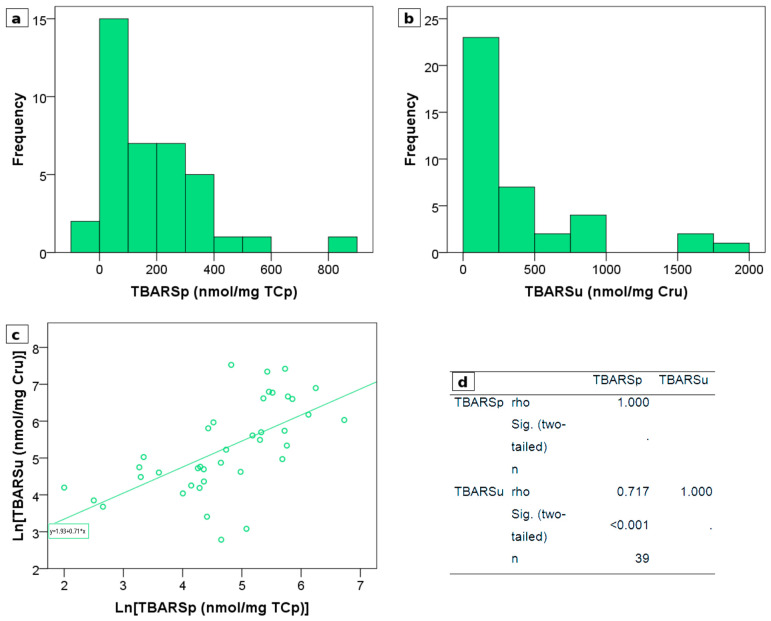
Distribution and association of plasma and urinary TBARS. (**a**,**b**) Histograms showing the distribution of cholesterol-normalized plasma TBARS (TBARSp) and creatinine-normalized urinary TBARS (TBARSu), respectively, both exhibiting non-normal distributions. Median and interquartile range values are provided in Table 1. (**c**) Scatter plot of TBARSp and TBARSu after natural logarithmic transformation of both variables, shown only for visualization of the monotonic association. The regression equation corresponds to the log–log representation; the asterisk in the equation denotes multiplication and is not intended to imply linearity on the original scale. (**d**) Rank correlation analysis showing Spearman’s ρ = 0.717, *p* < 0.001. TBARS—Thiobarbituric acid reactive substances; TBARSp—Plasma TBARS normalized by plasma total cholesterol; TBARSu—Urinary TBARS normalized by urinary creatinine; TCp—Plasma total cholesterol; Cru—Urinary creatinine; Ln—Natural logarithm.

A total of 39 treated patients with essential hypertension were included. The median age was 64 years, and 22 participants (56.4%) were women. Diabetes mellitus, chronic kidney disease, and cardiovascular disease were present in 5 (12.8%), 5 (12.8%), and 4 (10.3%) participants, respectively. The general characteristics of the study population are shown in Table 1. Median cholesterol-normalized plasma TBARS and creatinine-normalized urinary TBARS were 114 nmol/mg TCp and 152 nmol/mg Cru, respectively. The distributions of both variables are shown in Figure 1a,b. No missing values were identified in the variables of interest. A small number of extreme values were winsorized to the 1.5 × IQR limits (two observations in TBARSp and two in TBARSu) prior to the final inferential analyses.

### 3.2. Plasma–Urine Association of TBARS

Cholesterol-normalized plasma TBARS and creatinine-normalized urinary TBARS showed a moderate-to-strong positive monotonic association (Spearman’s ρ = 0.717, *p* < 0.001), as shown in Figure 1c,d. This indicates that participants with higher plasma lipid peroxidation-related TBARS tended to show higher urinary TBARS after creatinine normalization.

Sensitivity analyses showed that the association between TBARSp and TBARSu remained consistent after excluding participants with chronic kidney disease, diabetes mellitus, current smoking, or alcohol intake. Notably, after excluding participants with CKD, the correlation remained moderate-to-strong and statistically significant (Spearman’s ρ = 0.757, *p* = 2.22 × 10^−7^, n = 34), indicating that the plasma–urine association was not driven by participants with reduced renal function. Similar results were observed in the other sensitivity subsets, as shown in Supplementary Table S1 and Figure S1.

### 3.3. Exploratory ROC Analysis

**Figure 2:**
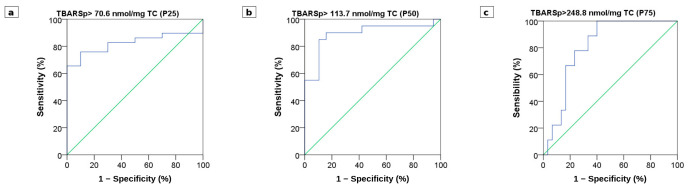
Exploratory ROC curve analyses of urinary TBARS for identifying higher plasma TBARS values. ROC curve models of creatinine-normalized urinary TBARS (TBARSu, nmol/mg Cru) for cholesterol-normalized plasma TBARS (TBARSp, nmol/mg TCp) using internally derived plasma percentile thresholds. (**a**) TBARSp > 70.6 nmol/mg TCp (P25): AUC = 0.824, SE = 0.066, *p* = 0.002, 95% CI: 0.696–0.953; sensitivity = 0.90, 1 − specificity = 0.20. (**b**) TBARSp > 113.7 nmol/mg TCp (P50): AUC = 0.892, SE = 0.057, *p* < 0.001, 95% CI: 0.781–0.999; sensitivity = 0.90, 1 − specificity = 0.16. (**c**) TBARSp > 248.8 nmol/mg TCp (P75): AUC = 0.811, SE = 0.068, *p* = 0.005, 95% CI: 0.678–0.944; sensitivity = 0.78, 1 − specificity = 0.23. ROC—Receiver operating characteristic; AUC—Area under the curve; SE—Standard error; CI—Confidence interval; TCp—Plasma total cholesterol; Cru—Urinary creatinine. The blue line represents the ROC curve of the model, and the diagonal green line represents the line of no discrimination (AUC = 0.5).

Exploratory ROC analyses were performed to assess whether creatinine-normalized urinary TBARS could discriminate participants with higher cholesterol-normalized plasma TBARS using internally derived percentile thresholds. Urinary TBARS showed within-cohort exploratory discriminatory capacity across the P25, P50, and P75 plasma TBARS thresholds. For the median-based threshold, urinary TBARS achieved an AUC of 0.892, with 90% sensitivity and 84% specificity. The results for the P25, P50, and P75 cut-off points are shown in Figure 2. Because these thresholds were derived from the internal distribution of the present cohort, these analyses should be interpreted as exploratory and not as evidence of externally validated diagnostic performance.

### 3.4. Bayesian Bootstrap Inference

**Figure 3:**
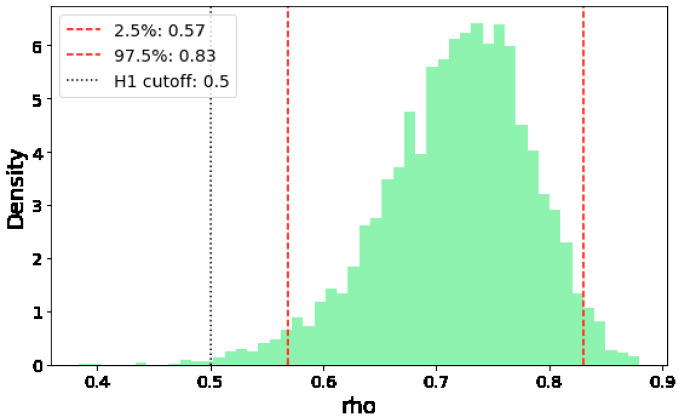
Posterior distribution of Spearman’s rank correlation coefficient (ρs) estimated via Bayesian bootstrap. The histogram represents 5000 bootstrap replications using Dirichlet-distributed weights applied to the original rank-transformed data. Red dashed lines indicate the 2.5th and 97.5th percentiles, corresponding to the 95% credible interval. The dotted black line marks the predefined threshold ρs = 0.5. The observed Spearman correlation was 0.717, with a 95% credible interval of 0.57–0.83 and BF10 = 332.33 for H1: ρs ≥ 0.5 versus H0: ρs < 0.5.

Bayesian bootstrap analysis yielded a posterior distribution centered around the observed Spearman correlation (ρs = 0.717), as illustrated in Figure 3. The 95% credible interval ranged from 0.57 to 0.83, supporting a moderate-to-strong monotonic association. The estimated Bayes factor comparing H1: ρs ≥ 0.5 versus H0: ρs < 0.5 was 332.33, indicating strong evidence that the true correlation exceeds 0.5.

## 4. Discussion

### 4.1. Main Finding and Novelty

In this paired observational study of treated patients with essential hypertension, creatinine-normalized urinary TBARS showed a moderate-to-strong positive monotonic association with cholesterol-normalized plasma TBARS. This relationship was supported by conventional rank-based correlation analysis and by Bayesian bootstrap inference, with a posterior credible interval consistent with a moderate-to-strong association. To our knowledge, this is the first paired-sample study specifically evaluating the relationship between cholesterol-normalized plasma TBARS and creatinine-normalized urinary TBARS in treated essential hypertension. Although previous studies have assessed plasma or urinary lipid peroxidation markers in different clinical contexts, these matrices have not usually been examined as paired, matrix-normalized readouts within the same individuals. In this sense, the present study forms part of a broader effort to systematically evaluate the plasma–urine relationship of redox biomarkers. Our group has recently applied this paired-matrix approach to reduced thiols in essential hypertension [39], whereas the present work extends it to TBARS as an operational marker of lipid peroxidation-related oxidative damage. These findings provide hypothesis-generating evidence that urinary TBARS may capture a lipid peroxidation-related signal aligned with plasma TBARS under standardized preanalytical conditions.

The relevance of this finding lies not in presenting urinary TBARS as a definitive clinical marker, but in establishing a measurable relationship between two accessible matrices. Plasma remains a privileged compartment for assessing vascular oxidative processes, particularly in hypertension, where oxidative stress arises within the same vascular environment in which circulating biomarkers are measured [3,4]. Urine, by contrast, offers a low-burden excretory matrix that may integrate part of this systemic oxidative signal over time [27]. Demonstrating that both normalized readouts move together within the same individuals is therefore a necessary first step toward developing urinary TBARS as an exploratory non-invasive proxy of plasma lipid peroxidation-related redox alterations.

### 4.2. Interpretation of the TBARS Plasma–Urine Association

The central interpretative point of this study is that urine appears to preserve information about a plasma lipid peroxidation-related signal, which is the compartment of greatest interest from a cardiovascular perspective. Plasma is directly exposed to vascular oxidative–inflammatory processes, including lipoprotein oxidation, endothelial dysfunction, and aldehyde generation in blood-facing vascular microenvironments. Urine, in contrast, is a downstream excretory matrix generated after glomerular filtration, tubular handling, urinary concentration, and variable residence time. Therefore, urinary TBARS would not necessarily be expected to closely parallel plasma TBARS. The fact that a moderate-to-strong monotonic association was observed despite these intervening biological layers is one of the most relevant findings of the present study.

This association should not be interpreted as evidence that urinary TBARS are a passive filtrate of plasma TBARS. Rather, it suggests that both matrices share a common systemic component, plausibly related to the circulating aldehydic burden generated by lipid peroxidation. Lipid peroxidation produces low-molecular-weight reactive products, including MDA-related aldehydes, which may circulate in free, protein-bound, metabolized, or conjugated forms and may subsequently contribute to urinary TBA-reactive signals after renal processing and excretion [6,25,27]. In this framework, the correlation between plasma and urinary TBARS may reflect a shared systemic peroxidative pressure, whereas the dispersion around the association may arise from renal handling, aldehyde metabolism, dilutional dynamics, local renal or urinary tract oxidative processes, and differences in urinary residence time.

Thus, to our knowledge, the novelty of this study lies in providing the first direct paired-sample evidence that urinary TBARS can track plasma TBARS in a chronic vascular condition characterized by oxidative–inflammatory stress when biologically grounded normalization strategies are applied to each matrix. This relationship became apparent after applying biologically grounded normalization strategies: plasma TBARS were normalized to total cholesterol to account for the lipid substrate context, whereas urinary TBARS were normalized to creatinine to reduce dilution-related variability. Therefore, the finding is not only that urinary TBA-reactive products are detectable, but that, when appropriately normalized, they show a consistent intra-individual association with the plasma compartment of greatest cardiovascular relevance. This supports the use of urine as a practical candidate matrix for estimating plasma lipid peroxidation-related redox alterations, while recognizing that the urinary signal is a biologically processed counterpart, rather than an identical replica, of the plasma signal.

### 4.3. Normalization and Analytical Interpretation

Normalization is central to the interpretation of the plasma–urine relationship observed in this study. Plasma TBARS were normalized to total cholesterol because cholesterol provides a routinely available proxy of the circulating lipid milieu in which lipid peroxidation products arise, particularly through lipoprotein-associated and vascular lipid-rich compartments [14,40]. This adjustment does not imply that cholesterol itself is the only or main oxidized substrate, but rather that total cholesterol can function as a practical denominator reflecting, at least in part, the lipid substrate context that conditions the generation and concentration of TBA-reactive products. In parallel, urinary TBARS were normalized to creatinine to reduce dilution-related variability, as commonly performed for urinary biomarkers [27,29]. Together, these corrections allowed comparison between two matrices with markedly different composition and concentration dynamics.

Importantly, these normalization strategies are not merely technical adjustments, but part of the biologically grounded signal-extraction strategy explored in this study. Alternative ways of expressing TBARS may obscure the plasma–urine relationship, whereas the combination of cholesterol normalization in plasma and creatinine normalization in urine helped reveal a measurable intra-individual association across the 39 paired samples. This supports the idea that the observed correlation is not simply an analytical coincidence, but may reflect a lipid peroxidation-related signal that becomes visible when each matrix is corrected according to a major source of variability: lipid substrate context in plasma and dilutional concentration in urine. A further strength of these correction factors is their routine clinical availability, since both total cholesterol and urinary creatinine are commonly obtained in standard laboratory assessments.

Nevertheless, both strategies remain pragmatic rather than definitive. Total cholesterol does not account for lipoprotein subclass distribution, PUFA content, lipid-lowering treatment, or differences in oxidative susceptibility across lipid compartments. Similarly, creatinine normalization may be influenced by renal function, muscle mass, age, sex, and tubular handling [29]. For this reason, renal function is particularly relevant when interpreting creatinine-normalized urinary TBARS, and its potential influence on the plasma–urine association was specifically addressed through sensitivity analyses. Future studies comparing alternative correction strategies, including lipoprotein-specific normalization, PUFA-related indices, urinary osmolality, specific gravity, and renal function-adjusted models, may help refine the interpretation of urinary TBARS.

### 4.4. Biological Plausibility in Hypertension and Oxidative–Inflammatory Vascular Disease

The observed plasma–urine association is particularly plausible in essential hypertension, a condition in which oxidative stress is closely intertwined with vascular dysfunction and low-grade inflammatory activation. Increased ROS production in hypertension arises from multiple sources, including NADPH oxidases, mitochondrial dysfunction, endothelial nitric oxide synthase uncoupling, renin–angiotensin–aldosterone system activation, and mechanical stretch imposed on the vascular wall [3,4]. These processes reduce nitric oxide bioavailability, promote endothelial dysfunction, amplify vascular remodeling, and favor redox-sensitive inflammatory signaling, thereby creating a biological setting in which lipid peroxidation products may be continuously generated within or near the circulating compartment [3,13,14].

Within this oxidative–inflammatory vascular environment, lipid peroxidation products are not merely passive markers of damage. Reactive aldehydes such as MDA- and 4-HNE-related species can modify proteins and lipoproteins, alter their biological behavior and contribute to endothelial activation, immune cell recruitment, and vascular dysfunction [6,8,9,10,14]. Therefore, the plasma TBARS signal detected in this study is biologically coherent with the pathophysiological context of treated essential hypertension. The fact that this plasma-related signal can also be detected in urine after creatinine normalization supports the possibility that urinary TBARS may serve as a low-burden candidate readout of lipid peroxidation-related oxidative–inflammatory activity, while remaining an indirect and operational biomarker rather than a direct measure of inflammation itself.

### 4.5. Translational Implications of Urine as a Matrix

From a translational perspective, the main value of the present finding is that urine may provide access to plasma-related lipid peroxidation information through a non-invasive and low-burden matrix. Plasma remains highly informative for vascular redox assessment, but its use is constrained by venipuncture, immediate processing requirements, centrifugation, cold-chain logistics, and limited suitability for repeated population-scale sampling. In contrast, urine can be collected easily, repeatedly, and with minimal discomfort, making it particularly attractive for longitudinal monitoring, epidemiological studies, and biomarker panels intended for broader cardiovascular risk assessment [27,28,39].

The present data do not establish urinary TBARS as a standalone diagnostic tool, nor do they define clinical thresholds for individual decision-making. Rather, they support urinary TBARS as a candidate exploratory proxy that may complement plasma-based redox assessment and conventional cardiovascular risk variables. This is especially relevant because the normalization factors used here, total cholesterol and urinary creatinine, are routinely available in standard clinical biochemistry, facilitating potential implementation without requiring specialized correction procedures. In future studies, urinary TBARS could be integrated with other redox biomarkers, renal parameters, inflammatory markers, vascular imaging readouts, and clinical outcomes to evaluate whether non-invasive redox profiling improves risk stratification, therapeutic monitoring, or mechanistic phenotyping in hypertension and related cardiometabolic conditions.

### 4.6. Statistical Support and Exploratory Discrimination

The statistical strategy used in this study provides convergent support for the plasma–urine association. Spearman’s rank correlation was appropriate for the non-normal distribution of both TBARS variables and showed a moderate-to-strong monotonic relationship between cholesterol-normalized plasma TBARS and creatinine-normalized urinary TBARS. The Bayesian bootstrap analysis further supported this interpretation by providing a distribution-free estimate of uncertainty around the correlation coefficient. The posterior distribution was centered around the observed association, and the credible interval remained compatible with a moderate-to-strong correlation, while also reflecting the uncertainty expected in a paired pilot study of this size [42,43,44,45].

The ROC analyses provide complementary, but exploratory, evidence. Urinary TBARS showed discriminatory capacity for identifying individuals with higher plasma TBARS across internally derived percentile thresholds, particularly for the median-based classification. However, these analyses should not be interpreted as establishing diagnostic thresholds or clinical decision limits. Because the P25, P50, and P75 thresholds were derived from the internal distribution of the same cohort, the resulting AUC values reflect within-cohort discrimination and are not directly comparable to diagnostic AUCs based on externally validated thresholds. Their value here is mainly to show that the plasma–urine association is not limited to a single correlation coefficient but is also reflected in the ability of urinary TBARS to stratify participants according to higher or lower plasma TBARS values within this exploratory dataset.

### 4.7. Limitations and Strengths

Several limitations should be acknowledged. First, this was a cross-sectional paired study based on a single sampling time point, which does not allow assessment of temporal stability, within-person tracking, responsiveness to clinical change, or prognostic value. Second, the sample size was modest and restricted to treated patients with essential hypertension, limiting generalizability to untreated hypertension, healthy individuals, advanced chronic kidney disease, or other inflammatory and cardiometabolic conditions. Third, TBARS are operational and non-specific measures of TBA-reactive aldehydic or carbonyl-containing products, rather than molecule-specific quantifications of MDA. Future studies should cross-validate plasma and urinary TBARS against more specific lipid peroxidation markers, such as F_2_-isoprostanes, LC-MS-based MDA measurements, 4-HNE adducts, or oxidized lipid/lipoprotein markers [8,14,16,17].

Additional limitations are related to matrix-specific biology and normalization. Although creatinine correction reduces urinary dilution-related variability, urinary TBARS may still be influenced by renal function, tubular handling, muscle mass, age, sex, urinary residence time, and potential local renal or urinary tract oxidative processes [27,29]. In the present study, sensitivity analyses showed that the TBARSp–TBARSu association remained consistent after excluding participants with CKD (Spearman’s ρ = 0.757, *p* = 2.22 × 10^−7^, n = 34), indicating that the association was not driven by this subgroup. Nevertheless, larger studies should evaluate renal function more extensively, including eGFR-adjusted models and alternative urinary correction strategies.

Similarly, total cholesterol normalization provides a practical proxy of the circulating lipid milieu, but it does not account for lipoprotein subclass distribution, PUFA content, lipid-lowering treatment, or differences in oxidative susceptibility across lipid compartments. Finally, the ROC thresholds were internally derived from the present cohort and should not be interpreted as diagnostic cut-offs or clinical decision limits without external validation.

The study also has relevant strengths. The paired design minimized interindividual variability by comparing plasma and urine within the same participants. Blood and urine samples were obtained during the same on-site fasting visit and processed using rapid cold-controlled preanalytical procedures, including immediate cooling, centrifugation, aliquoting, dry-ice transfer, and storage at −80 °C. Plasma and urine were analyzed under standardized analytical conditions, reducing methodological asymmetry between matrices. A further strength is that the plasma–urine association was evaluated using biologically grounded and routinely available normalization factors, allowing a matrix-specific comparison between the plasma lipid substrate context and urinary dilutional correction. Finally, the convergence of Spearman correlation, exploratory ROC analysis, Bayesian bootstrap inference, and renal-function sensitivity analyses provides internally consistent statistical support for the observed plasma–urine relationship.

## 5. Conclusions

In treated patients with essential hypertension, creatinine-normalized urinary TBARS showed a moderate-to-strong intra-individual association with cholesterol-normalized plasma TBARS under standardized paired-sample conditions. This finding suggests that urine can preserve a biologically processed counterpart of the plasma lipid peroxidation-related signal, despite the renal and excretory processes separating both matrices. The study therefore provides hypothesis-generating evidence supporting urinary TBARS as a practical, low-burden, and non-invasive candidate proxy for plasma lipid peroxidation-related redox alterations in a vascular oxidative–inflammatory context.

Importantly, the observed association became apparent using normalization factors that are biologically grounded and routinely available in clinical laboratories: total cholesterol in plasma and creatinine in urine. This supports the potential translational relevance of the approach, while also emphasizing the need for further validation. Larger, independent, and clinically diverse cohorts should assess the longitudinal stability, responsiveness to clinical change, and prognostic value of urinary TBARS, ideally incorporating more specific lipid peroxidation markers, cardiovascular outcome measures, and renal-function-aware validation strategies.

## 6. Patents

The authors report that part of the methodology used in this work is included in a European patent application filed by the Servizo Galego de Saúde, Universidade de Santiago de Compostela and IDIS. The patent application “IN VITRO METHOD FOR ASSESSING OXIDATIVE STRESS” was filed at the European Patent Office on 22 April 2025 under application number EP25382404.9 (submission number 300560808). The inventors listed are Antón Cruces-Sande, Néstor Vázquez-Agra, Álvaro Hermida-Ameijeiras, Estefanía Méndez-Álvarez and Ramón Soto-Otero.

## Figures and Tables

**Table 1:** Clinical, biochemical, and redox-biomarker characteristics of the study population.

Variables	Total
	*n* = 39
Age (years) †	64 (19)
Sex (women) ‡	22 (56.4)
Alcohol intake ^a^ ‡	6 (15.4)
Smokers ^b^ ‡	5 (12.8)
Physical activity ^c^ ‡	17 (43.6)
Diabetes ^d^ ‡	5 (12.8)
CKD ^e^ ‡	5 (12.8)
CVD ^f^ ‡	4 (10.3)
BMI (kg/m^2^) †	28 (7)
SBP (mmHg) †	128 (19)
DBP (mmHg) †	74 (11)
HR (bpm) †	68 (11)
Antihypertensive drugs ‡	31 (79.5)
RAAS blockers ‡	26 (67)
Plasma creatinine (mg/dL) †	0.79 (0.3)
TCp (mg/dL) †	188 (48)
TBARSp (nmol/mg TCp) †	114 (178)
Cru (mg/dL) †	132 (71)
TBARSu (nmol/mg Cru) †	152 (404)

^a^ Low-risk alcohol consumption defined as <10 g/day for women and <20 g/day for men. ^b^ Current smokers or those who smoked within the previous 6 months. ^c^ Moderate physical activity equivalent to 150 min of moderate-intensity walking per week. ^d^ Diabetes defined according to ADA criteria. ^e^ CKD defined as eGFR < 60 mL/min/1.73 m^2^. ^f^ CVD defined according to ESC criteria. Results expressed as † refer to median and interquartile range, and results expressed as ‡ refer to number and percentage. CKD—Chronic kidney disease; CVD—Cardiovascular disease; BMI—Body mass index; SBP—Systolic blood pressure; DBP—Diastolic blood pressure; HR—Heart rate; RAAS—Renin–angiotensin–aldosterone system; TCp—Plasma total cholesterol; TBARS—Thiobarbituric acid reactive substances; TBARSp—Plasma TBARS normalized by plasma total cholesterol; Cru—Urinary creatinine; TBARSu—Urinary TBARS normalized by urinary creatinine.

## Data Availability

The data supporting the findings of this study are available from the corresponding author upon reasonable request. Public deposition is not possible due to patient confidentiality constraints.

## Supplementary Materials

The following supporting information can be downloaded at: https://www.mdpi.com/article/10.3390/antiox15070861/s1, Supplementary Figure S1. Sensitivity analysis of the Spearman correlation between plasma and urinary TBARS. Scatterplot showing the relationship between natural logarithm-transformed cholesterol-normalized plasma TBARS (TBARSp) and creatinine-normalized urinary TBARS (TBARSu) among participants with complete paired measurements. Lines represent fitted trends for the overall sample and for each sensitivity subset. The overall Spearman correlation was ρ = 0.717, *p* = 2.52 × 10^−7^. The correlation remained consistent after excluding current smokers, participants reporting alcohol intake, patients with chronic kidney disease (CKD), and patients with diabetes. TBARS—thiobarbituric acid reactive substances; TBARSp—plasma TBARS normalized by plasma total cholesterol; TBARSu—urinary TBARS normalized by urinary creatinine; CKD—chronic kidney disease. Supplementary Table S1. Sensitivity analysis of Spearman’s rank correlation between urinary and plasma TBARS. ρ denotes Spearman’s rank correlation coefficient. Valid *n* denotes the number of participants with complete paired urinary/plasma TBARS measurements included in each analysis. Each sensitivity analysis excluded participants with the corresponding exposure or condition. Cases with unknown smoking or alcohol status were excluded from the corresponding sensitivity analysis. TBARS—thiobarbituric acid reactive substances; CKD—chronic kidney disease.

## Abbreviations

ADA	American Diabetes Association
AUC	Area under the curve
BF10	Bayes factor for the alternative hypothesis versus the null hypothesis
BHT	Butylated hydroxytoluene
BMI	Body mass index
CHUS	University Hospital Complex of Santiago de Compostela
CI	Confidence interval
CKD	Chronic kidney disease
Cru	Urinary creatinine
CVD	Cardiovascular disease
DBP	Diastolic blood pressure
EDTA	Ethylenediaminetetraacetic acid
eGFR	Estimated glomerular filtration rate
ESC	European Society of Cardiology
ESH	European Society of Hypertension
F_2_-IsoPs	F_2_-isoprostanes
GCP	Good Clinical Practice
4-HNE	4-Hydroxy-2-nonenal
HR	Heart rate
IDIS	Health Research Institute of Santiago de Compostela
ISCIII	Instituto de Salud Carlos III
IQR	Interquartile range
LC-MS	Liquid chromatography–mass spectrometry
MDA	Malondialdehyde
NADPH	Nicotinamide adenine dinucleotide phosphate
PUFA	Polyunsaturated fatty acid
RAAS	Renin–angiotensin–aldosterone system
ROC	Receiver operating characteristic
ROS	Reactive oxygen species
SBP	Systolic blood pressure
SE	Standard error
TBA	Thiobarbituric acid
TBARS	Thiobarbituric acid reactive substances
TBARSp	Plasma TBARS normalized by plasma total cholesterol
TBARSu	Urinary TBARS normalized by urinary creatinine
TCp	Plasma total cholesterol
ρ	Spearman’s rho
ρs	Spearman’s rank correlation coefficient
