# Supplementary material for: Urinary TBARS as a Non-Invasive Proxy of Plasma Lipid Peroxidation in Essential Hypertension: A Translational Study on Vascular Oxidative–Inflammatory Burden
Source: Antioxidants (Basel). 2026 Jul 9;15(7):861. doi: 10.3390/antiox15070861 (PMC13403372; doi:10.3390/antiox15070861)
Supplement: Supplementary file 1 [file antioxidants-15-00861-s001.zip › antioxidants-4361862-supplementary.pdf]

Supplementary Table S1. Sensitivity analysis of Spearman's rank correlation between urinary and plasma TBARS.

Analysis	Valid n	Spearman rho	p-value
Overall	39	0.717	$2.52×10^{-7}$
Excluding smokers	32	0.701	$8.02×10^{-6}$
Excluding drinkers	31	0.722	$4.58×10^{-6}$
Excluding CKD	34	0.757	$2.22×10^{-7}$
Excluding diabetes	34	0.760	$1.81×10^{-7}$

rho denotes Spearman's rank correlation coefficient. Only complete urinary/plasma TBARS pairs were analyzed. Each sensitivity analysis excluded participants with the corresponding exposure or condition. Cases with unknown smoking or alcohol status were excluded from the corresponding sensitivity analysis. CKD, chronic kidney disease.

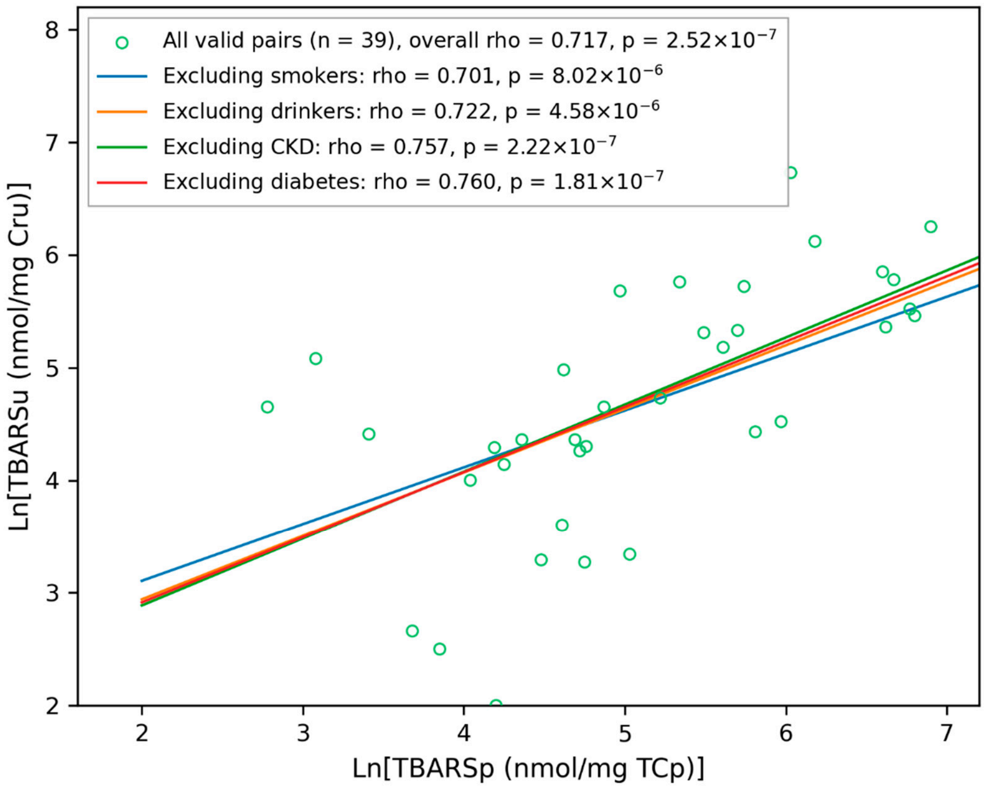

Supplementary Figure S1. Sensitivity analysis of the Spearman correlation between plasma and urinary TBARS. Scatterplot showing the relationship between ln-transformed plasma TBARS and urinary TBARS among participants with complete paired measurements. Lines represent fitted trends for each sensitivity subset. The overall Spearman correlation was rho = 0.717, $p = 2.52×10^{-7}$. The correlation remained consistent after excluding smokers, alcohol users, patients with chronic kidney disease (CKD), and patients with diabetes.
